# A Look at Cycling Safety in a Southern Municipality of the Netherlands

**DOI:** 10.7759/cureus.25268

**Published:** 2022-05-24

**Authors:** Peter O Onaiyekan, Valéria L Passos, Klasien Horstman

**Affiliations:** 1 General Practice, Namoi Medical Services, Narrabri, AUS; 2 CAPHRI (Care and Public Health Research Institute) Faculty of Health, Medicine & Life Sciences, Maastricht University, Maastricht, NLD

**Keywords:** safety, netherlands, maastricht, data analysis, cycling, accidents

## Abstract

Objectives

Cycling is an important means of transportation in the Netherlands. Unfortunately, the number of cycling accidents and their adverse outcomes (injury and death) are on the rise. We set out to observe the nature of these accidents in Maastricht from 2001 to 2015 and analyzed the recommendations of stakeholders on ways to improve cycling safety.

Methods

An explanatory sequential mixed methodology was used for this population-based study. In the first phase, a retrospective quantitative analysis of the VIA® accident database for Maastricht was done. This was followed by a thematic analysis of data from five semi-structured interviews. Integration was at the Interpretation stage.

Result

The first phase showed males (54%) and under-25s (59.9%) had the most cycling accidents, while a larger percentage of females (50.7%) and people >65 years (67%) had adverse outcomes with accidents. More accidents occurred at intersections (52.6%), on shared roads (61.4%), and involved motorized vehicles (95.6%). Bivariate analysis and multivariable logistic regression showed that cycling accidents involving elderly people, women, wet weather or road surfaces, an innocent cyclist, the northeastern district, and morning hours had a higher chance of injury or death. Thematic analysis summarised stakeholder opinions under four themes: role in cycling safety; partners of cycling safety; the importance of accurate data; and investing in safety. Most of the respondents felt improvements in the city’s accident database, cycling policy, and infrastructure were needed.

Conclusion

Our findings suggest that there has been a decrease in the overall number of cycling accidents in the 15-year period studied. However, differences in sociodemographic variables still determine the distribution and severity of accidents in Maastricht. The existing cycling database at the time of the study needed improvements with data collection and the city needs to involve more stakeholders in its policy-making process.

## Introduction

In the world today, most injuries and fatalities from Road Traffic Accidents (RTAs) are in low-and-middle-income countries (LMICs). Yet, RTAs still occur in more advanced countries where, like anywhere else in the globe, they are a cause of immense sorrow and suffering [1-4]. The Netherlands has a low record of RTAs and fatalities bested only by countries like Sweden, Switzerland, and the United Kingdom among a few others [5]. Programs like Sustainable Safety Vision (Duurzam veilig), launched in the early 1990s as a collaboration between the Institute for Road Safety Research and the Dutch Central Government [6,7], have played a big role in guiding policy. The Sustainable Safety Vision had two objectives: preventing crashes from occurring and preventing severe injury if a crash could not be avoided [8].

Of growing importance in the Netherlands, now, is cycling safety due to a steady increase in the numbers and severity of cycling accidents when compared to other road vehicles [9-12]. Currently, there are almost as many cycling fatalities - about 200 cyclists killed each year since 2009 - as there are amongst car users [11,13]. This rise is mainly driven by deaths among men aged 65 and above who use electric bikes and made up three-quarters of the 206 cyclists killed in 2017 [14].

With about 16.5 million inhabitants, an estimated 18 million bicycles, and about 35,000 km of cycle paths, each person in the Netherlands can cycle an average distance of 1000 km each year [15-17], making it, along with Denmark, one of the top two leading cycling nations in Europe [18,19]. The combination of these numbers with the relative rise in fatalities and serious injuries among the economically vibrant youthful age groups and the elderly portends a growing public health issue [5,9,10,12]. Indeed, both the Netherlands and Denmark were shown to have had the highest percentage of cyclist fatalities in 2008 with the Netherlands having about 21% and Denmark about 13% [20]. As such, “investigating the problem of increasing cycling injuries and fatalities provides an opportunity to improve the safety of this cheap, convenient and environmentally safe mode of transport” [20].

Although cycling safety in the Netherlands falls under the purview of authorities like the Ministry of Infrastructure and the Environment - which is currently looking into ways to regulate E-bike use among the elderly and phone use while cycling among the young [21,22] - it is majorly the autonomous local governments (municipalities) that are responsible for maintaining infrastructure, widening and improving cycle paths, and who really promote cycling safety in the country [13,14,23,24]. These Dutch municipalities and cities are built to be compact and have nearly half of all trips made in them being below 2.5 km, a situation that is ideal for the development of cycling [12,25]. Maastricht is a quintessential municipality, being an old, compact city with a high degree of urbanization, several cycling facilities, and topographic features attractive for cycling [26-30].

And so, we set out to assess the general trend and patterns of cycling accidents in Maastricht from 2001 to 2015 and to investigate risk factors for cycling injuries and deaths over this time span. Our ultimate goal was to evaluate how the obtained empirical findings could contribute to future policies while assessing the effectiveness of cycling accident data collection. Understanding trends in the city, during this period, was relevant as it was an era that witnessed the commencement of two major cycling safety plans, namely, the Strategic Plan for Road Safety [6] and the Safe Cycling Network project [13], thereby helping monitor their implementation in Maastricht.

We also sought to understand the perceptions of major cycling stakeholders on the identified trends, the quality of data, and future cycling policies. Stakeholder analysis has been shown to generate information about important actors in a field of endeavor, thereby, “understanding their behavior, intentions, interrelations, agendas, interests, and the influence or resources they have brought - or could bring - to bear on decision-making processes” [31].

Thus, the study utilized both a systematic approach to analyzing data and stakeholder opinions in understanding the nature of cycling accidents in Maastricht, incorporating the principles of quantitative and qualitative research.

## Materials and methods

We combined data from Maastricht city's approved traffic accident database, called VIA® Traffic Solutions Software (Vught, the Netherlands) database, with the everyday experiences of different working groups using the concept of Complementarity [32] and an Explanatory Sequential design. Both quantitative and qualitative findings were then integrated at the interpretation (discussion) stage of the research.

Data collection

Quantitative

Retrospective data on cycling accidents in the city of Maastricht, from 2001 to 2015, were obtained from the VIA online database (http://www.via.nl). This database is a product of collaboration between the Dutch police, the Dutch Association of Insurers, and VIA traffic data specialists [33]. It contains details of accidents known to the Dutch police or insurance companies.

A total of 1656 cycling accident victims were recorded in the database for Maastricht. We included all cycling accidents which occurred between January 2001 and December 2015, leaving us with the following variables: accident severity; type of injury sustained; the types of vehicles involved; whether the cyclist was at fault or not; the main cause of the accident; the time period of the accident; the day; the month; the district; the part of the road involved; the weather, state of the road, and light condition; and type of road on which cyclist was riding.

The outcome measure, accident severity, was operationalized as 0=no injuries and 1=injuries, with 1 consisting of cases classified as “type of injury sustained” - mild, severe, and fatal. The variable month was recoded into seasons and used for analysis that way. Cases occurring in December, January, and February made up Winter (1); March, April, and May - Spring (2); June, July, and August - Summer (3); and September, October, and November - Autumn (4). Age was recoded into a categorical variable Age group- from 0 to 4 (0= <12 years, 1= 12-18 years, 2= 18-25 years, 3= 25-65 years, and 4= >65 years). Also, the different points of the road involved in an accident were recoded into three categories - straight road (0), intersection (1), and roundabout (2). Finally, the variable “time period of the accident” was recoded into three categories: Morning (0), Afternoon (1), and Evening/Night (2).

We excluded all variables with more than 25% missing values like street name, alcohol use by non-cycling parties, and drug use by the cyclist or not, among others. Accidents not involving a cyclist as well as those which occurred outside of Maastricht were also excluded.

Qualitative

This phase involved seven semi-structured interviews with informants in the field of road traffic safety at the Gemeente Maastricht (Municipality), Fietsersbond (Dutch Cyclists’ Union), SWOV (Institute for Road Safety Research), Maastricht ambulance services, and the Politie (Police). The organizations visited were important stakeholders in road traffic safety and had policies and action plans on cycling safety. In turn, the informants within these organizations were chosen specifically for their experience and versatile knowledge of cycling issues in Maastricht.

The semi-structured interviews were done in English, a language that all informants were proficient in, and they lasted between forty minutes to an hour. They were mostly face-to-face interviews, except for one occasion where it occurred via Skype®. The questions asked (see Appendices) were broadly grouped into four sets: questions about the informant’s organization and the policies on cycling of importance to them; questions on the organization’s partners for safe cycling in Maastricht; questions about data collection on cycling accidents in Maastricht and their perceptions of the first phase quantitative analysis; and finally, questions about their opinions on how to improve cycling safety in Maastricht.

Quantitative data analysis

The data collected from VIA® statistics was transferred into SPSS (Statistical Package for the Social Sciences) version 20.0 (IBM Corp., Armonk, USA) for analysis. Descriptive statistics with the distribution of variables based on demographics (age - continuous, age group - categorical, and sex), physical characteristics (weather, season, time of day, month, year, state of the road, light condition, type of road), and accident circumstances (alcohol use by a cyclist, cyclist at fault or not, accident severity, type of injuries sustained, type of vehicles involved, and main cause).

Logistic regression was used to investigate links between the independent variables and the dichotomous outcome of accidents with injuries. Visual inspection of the data suggested that the proportion of the latter changed over time in a nonlinear fashion. Therefore, polynomials (Year2 and Year3) were created to model the temporal pattern of injuries over time. Odds ratios (OR), and 95% confidence intervals (CI) were significant at a p-value of < 0.05.

Qualitative data analysis

For the qualitative component, each interview was recorded and then transcribed. Each transcript was subsequently explored using thematic analysis. Themes were derived from the questions asked, and then coding of keywords and phrases was done to create categories. The categories from each organization were then tabulated and compared to identify areas of similarities or differences.

Ethical considerations

Maastricht University declared that ethical approval was not applicable for this study. Permission to access the VIASTAT database was granted by the Gemeente Maastricht and VIA, and informed consent was obtained from all informants for the semi-structured interviews. Care was taken to ensure the anonymity of all participants and data was handled in a secured manner.

## Results

Quantitative

Descriptive Statistics

**Figure 1:**
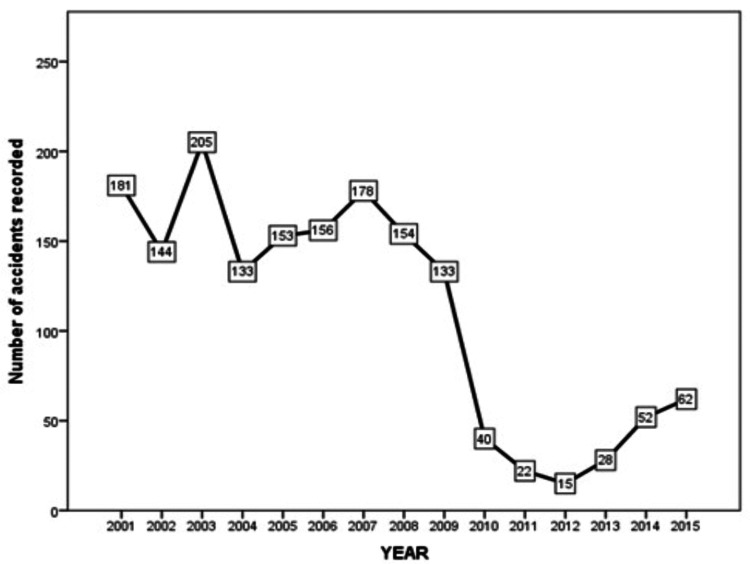
Line graph showing the number of cycling accidents in Maastricht from 2001 to 2015.

In 2003, cycling accidents peaked in Maastricht, with a 70% drop noted in the numbers between 2009 and 2010 (Figure 1).

**Figure 2:**
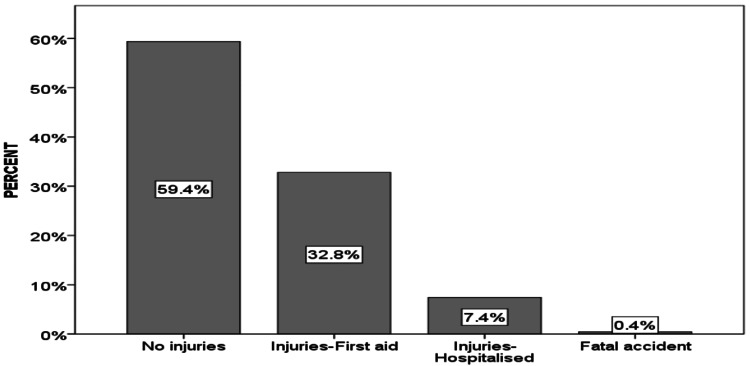
Percentage distribution of injuries sustained in cycling accidents in Maastricht.

Most of the accidents were injury-free or minor (92.2%, n=1515) with only 7.8% being fatal or requiring some sort of hospitalization (Figure 2).

The severity of a cycling accident varied depending on the variable analyzed. Table 1 summarizes the observed effects.

**Table 1:** Crash level (severity) of cycling accidents in Maastricht by sociodemographic, physical and behavioural variables (2001-2015).

Variable		Crash level					
		No injuries		Injuries		OR	p-value
		Frequency	%	Frequency	%		
Age group	Older than 65 years	39	36.8	67	63.2	1.000	<0.001
	Younger than 12 years	146	74.9	49	25.1	0.195	<0.001
	12-18 years	243	69.0	109	31.0	0.261	<0.001
	18-25 years	192	65.5	101	34.5	0.306	<0.001
	25-65 years	354	50.9	342	49.1	0.562	0.007
Sex	Male	553	64.4	307	35.6	1.000	
	Female	384	52.0	355	48.0	1.671	<0.001
Weather	Dry	859	60.6	558	39.4	1.000	
	Wet	99	49.5	101	50.5	1.571	0.003
Parties involved	Bicycle only or with Pedestrian	33	49.3	34	50.7	1.000	0.028
	Bicycle+Two wheeled vehicle	290	63.2	169	36.8	0.566	0.030
	Bicycle+Motorcar/Van	563	57.3	419	42.7	0.722	0.198
	Bicycle+Heavy vehicle	22	47.8	24	52.2	1.059	0.881
Type of road involved	Shared road	560	59.5	381	40.5	1.000	
	Bicycle lane	335	57.4	250	42.6	1.090	0.417
Road section involved	Roundabout	91	59.1	63	40.1	1.000	0.963
	Straight road	365	59.0	254	41.0	1.005	0.978
	Intersection	516	59.7	349	40.3	0.977	0.896
Season	Autumn	228	54.8	188	45.2	1.000	0.139
	Winter	209	63.9	118	36.1	0.810	0.154
	Spring	269	62.3	163	37.7	0.724	0.022
	Summer	205	57.4	152	42.6	0.837	0.216
Cyclist’s fault	Yes	442	62.2	256	37.8	1.000	
	No	550	57.3	410	42.7	1.229	0.045
District	Maastricht Central/Southwest	179	67.3	89	32.7	1.000	0.023
	Southeast	250	59.5	170	40.5	1.399	0.041
	Northwest	216	58.2	155	41.8	1.476	0.020
	Northeast	313	56.1	245	43.9	1.610	0.002
Time of week	Weekday	673	60.4	442	39.6	1.000	
	Weekend	301	57.4	223	42.6	1.128	0.262
Time of day	Evening/Night	194	62.4	117	37.6	1.000	0.063
	Morning	272	55.1	222	44.9	1.353	0.041
	Afternoon	507	60.6	329	39.4	1.076	0.592
State of road surface	Dry	795	61.2	503	38.8	1.000	
	Wet	166	51.4	157	48.6	1.495	0.001
Light status	Bright	794	59.3	546	40.7	1.000	
	Dark	173	58.6	122	41.4	1.026	0.847

**Figure 3:**
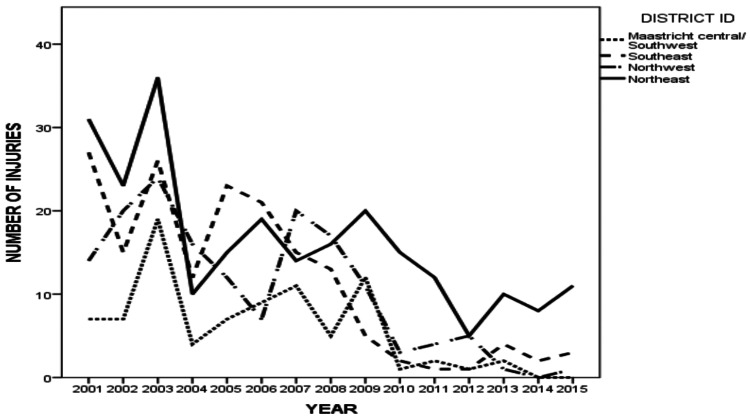
Temporal distribution of cycling injuries (absolute counts) in the different districts of Maastricht.

Based on regions, the Eastern districts were the most dangerous (northeast - 35% and southeast - 26%), however, there was a general decline in all districts with time (Figure 3).

Unadjusted Analysis

Bivariate analysis as seen in Table 2 of the appendices shows that the age group of an accident victim was important in determining the severity of an accident. All age groups less than 65 years had lower chances of injuries after an accident (<12 years - OR=0.195, CI=0.117-0.325; 12-18 years - OR=0.261, CI=0.166-0.411; 18-25 years - OR=0.306, CI=0.193-0.486; 25-65 - OR=0.562, CI=0.369-0.857). Females were more likely to have an adverse outcome than males (OR=1.671, CI=1.367-2.042). Cycling in wet weather resulted in accidents with a higher risk of injury when compared to dry weather (OR=1.571, CI=1.167-2.114); as was generally the case with cycling on a wet surfaced road compared to a dry one (OR=1.495, CI=1.170-1.910).

**Table 2:** Unadjusted analysis showing crude odds ratios of independent variables

Variables	p-value	OR	CI	
Sex				
Male		1.000		
Female	<0.001	1.671	1.367-2.042	
Age continuous				
Age	<0.001	1.024	1.019-1.030	
Age categorical				
Age: Older than 65	<0.001	1.000		
Age: Younger than 12	<0.001	0.195	0.117-0.325	
Age: 12-18	<0.001	0.261	0.166-0.411	
Age: 18-25	<0.001	0.306	0.193-0.486	
Age: 25-65	0.007	0.562	0.369-0.857	
Year continuous				
Year	0.001	0.642	0.492-0.836	
Year categorical				
Year 2001	<0.001	1.000		
Year 2002	0.812	1.055	0.678-1.642	
Year 2003	0.142	1.353	0.904 -2.025	
Year 2004	0.022	0.577	0.361-0.922	
Year 2005	0.184	0.742	0.478-1.153	
Year 2006	0.112	0.700	0.451-1.087	
Year 2007	0.042	0.641	0.418-0.984	
Year 2008	0.039	0.625	0.400-0.977	
Year 2009	0.138	0.706	0.446 -1.118	
Year 2010	0.356	1.382	0.695-2.745	
Year 2011	0.001	7.917	2.262-27.705	
Year 2012	0.015	5.000	1.364-18.326	
Year 2013	0.113	1.932	0.856-4.357	
Year 2014	0.084	0.551	0.281-1.083	
Year 2015	0.132	0.625	0.339-1.153	
Year polynomial				
Year^2^	0.004	1.063	1.020-1.108	
Year^3 ^	0.009	0.998	0.996-0.999	
Season				
Autumn	0.139	1.000		
Winter	0.154	0.810	0.607-1.082	
Spring	0.022	0.724	0.549-0.954	
Summer	0.216	0.837	0.631-1.110	
Time of week				
Weekday		1.000		
Weekend	0.262	1.128	0.914-1.393	
Time of day				
Evening/Night	0.063	1.000		
Morning	0.041	1.353	1.013-1.808	
Afternoon	0.592	1.076	0.823-1.407	
District				
Maastricht Central/Southwest	0.023	1.000		
Southeast	0.041	1.399	1.014-1.930	
Northwest	0.020	1.476	1.063-2.051	
Northeast	0.002	1.610	1.186-2.187	
State of road surface				
Dry		1.000		
Wet	0.001	1.495	1.170-1.910	
Light status				
Daylight		1.000		
Dark	0.847	1.026	0.794-1.325	
Weather				
Dry		1.000		
Wet	0.003	1.571	1.167-2.114	
Parties involved				
Bicycle-only or with Pedestrian	0.028	1.000		
Bicycle + Two wheeler	0.030	0.566	0.338-0.947	
Bicycle + Motorcar/Van	0.198	0.722	0.440-1.185	
Bicycle + Heavy vehicle	0.881	1.059	0.500-2.244	
Cyclist at fault				
Yes		1.000		
No	0.045	1.229	1.005-1.503	
Alcohol use				
Yes		1.000		
No	0.736	0.892	0.458-1.737	
Type of road involved				
Shared road		1.000		
Bicycle lane	0.417	1.090	0.885-1.344	
Road section involved				
Roundabout	0.963	1.000		
Straight road	0.978	1.005	0.702-1.439	
Intersection	0.896	0.977	0.689-1.385	

Regarding districts, accidents that occurred in Maastricht Central and Southwest had less chance of an injury when compared with those in the other three districts (Southeast - OR=1.399, CI=1.014-1.930; Northwest - OR=1.476, CI=1.063-2.051; and Northeast - OR=1.610, CI=1.186-2.187). Other variables with a greater risk of cycling injuries included accidents where the cyclist was not at fault, and those occurring in the morning (Cyclist not at fault - OR=1.229, CI=1.005-1.503; Morning - OR=1.353, CI=1.013-1.808).

Cycling accidents involving cyclists and other two-wheeled vehicles as well as spring season had less risk of injuries (Bicycle + two-wheeled vehicle - OR=0.566, CI=0.338-0.947; Spring - OR=0.724, CI=0.549-0.954).

Multivariable Analysis

All significant variables in the unadjusted analysis were added to the multivariable logistic regression model, which entailed sex, age (categorical), year (continuous) and its polynomials, time of day, district, weather, parties involved, and interaction between year and district of accident occurrence.

**Figure 4:**
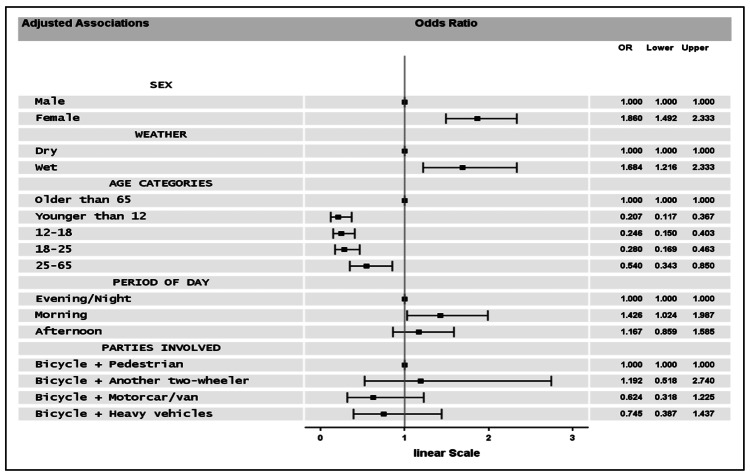
Forest plots showing the Odds Ratios of the independent effects in the final model.

Adjusted effects are presented in terms of ORs in Figure 4 for the variables showing an independent link to the outcome. Fewer odds of sustaining an adverse effect for cyclists aged below 65 years as against those older than 65 were observed. Sex, Weather, and Period of day variables had non-referent variables with higher - injuries were more likely in the morning, in wet weather, and for female cyclists. Age had the larger impact, as results indicate that the odds were substantially smaller for younger compared to the elderly (65+).

**Figure 5:**
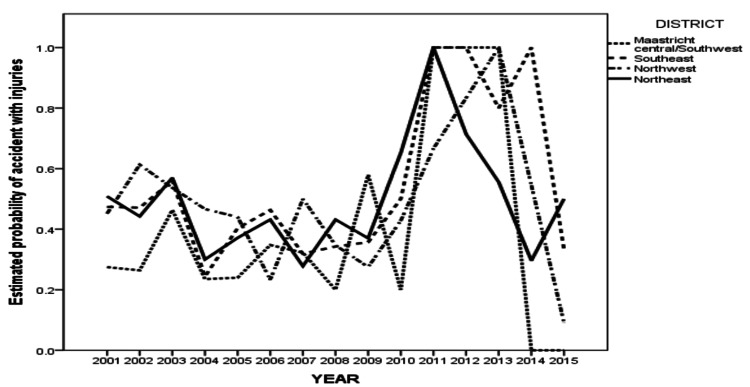
Plot showing differences in estimated probabilities of having an adverse outcome in a cycling accident in the districts of Maastricht as a function of the year.

A significant interaction between district and year indicates that either the difference in the likelihood for adverse outcomes, seen among districts, depended on the year of occurrence or that changes in the odds of injury over the years were not the same for all districts. To facilitate the interpretation of this interaction we present the estimated probabilities of having an adverse outcome, according to the multivariable logistic model in Figure 5.

The estimated likelihood of injuries over time steadily decreased for the northeast district between the years 2001-2007, whereas Maastricht central showed a different pattern, also decreasing in the first 4 years, but with a steady increase thereafter. This figure also shows a paucity of dots with instability of the graph from 2010. This was found to most likely be a result of reduced reporting.

Qualitative

The following themes: role in cycling safety; partners of cycling safety; importance of accurate data; and investing in safety were identified from thematic analysis.

Role in Cycling Safety

Most respondents said their contributions to cycling safety, involved primary prevention and centered on cycling-friendly policy creation and implementation, and infrastructural development.

We develop and design cycling infrastructure…and formulate cycling-friendly policies. (Municipality)

…We want more cyclists and need to have a safe and comfortable infrastructure to achieve this. (Cyclists’ Union)

We do research on road safety - road user behavior, infrastructure, research, and laws concerning epidemiology... (SWOV)

They also felt that an enabling environment for cycling depends on plans which are detailed and productive and which will result in more people choosing cycling as a regular mode of commute:

…*cyclist-friendly infrastructure leads to more cycling… (SWOV)*

Educating cyclists on policies and responsible road use was of importance to most organizations.

…we promote safety awareness and good behavior amongst cyclists… (Municipality)

We try to improve the education of new cyclists, especially amongst the young. (Cyclists’ Union)

We run educational programs on safe cycling and evaluations involving secondary school students… (SWOV)

While the police were primarily involved in enforcing laws:

We enforce laws on cycling safety, for example clamping down on drunken car drivers or cyclists and ensuring bikers use lights in the dark… (Police)

There is an agreement with the police on enforcing laws that improve road safety... (Municipality)

…the right to enforce most traffic laws lies with the police who are under the central government… (SWOV)

The ambulance service was involved in secondary prevention, being a first responder in serious accidents.

…no direct role to play in public health prevention of road or cycling accidents…We are involved only in treating injuries and not necessarily in taking precautions on the road or any other preventive measure… (Ambulance service)

Partners of Cycling Safety

All five organizations worked together on numerous occasions; however, their relationships were not always warm:

…the relationship between stakeholders is dynamic so there are moments when disagreements exist. (Municipality)

…our reservations about the bus lane were ignored at first, but a year later, the municipality has now agreed that it made the wrong decision… (Cyclists’ Union)

The interactions were widespread and diverse:

…using lots of contacts in the city council, and also in the media… Another organization we work with is the VVN (Veilig Verkeer Nederland - Safe Traffic Netherlands​​​​​​), it is mainly focused on promoting road safety, and good road behavior…neighborhood organizations, and companies. Sometimes there is cooperation with the police for educational purposes. (Cyclists’ Union)

*…Traumatologists who encourage helmet wearing…a group of psychologists working on road safety in the Netherlands…Fietsberaad (CROW - Centrum voor Regelgeving en Onderzoek in de Wegenbouw - Centre for Regulation and Research in Road Construction* *) - knowledge portal between research and practical applications, Fietsersbond - a cyclist lobby group…Provinces and Municipalities are also important partners. (SWOV)*

…the traffic office in the municipality…ROVL (Regionaal Orgaan Verkeersveiligheid Limburg - Limburg Road Safety Regional Body​​​​​​​) has programs that try to stop biking at night without lights… ANWB (Algemene Nederlandse Wielen Bond) - yellow cars - which started hundreds of years ago by creating safe zones for bicycles …the insurance companies… Stichting processen verbal (a foundation that registers accident data from the police). (Police)

We also work closely with the police who in most cases are the first to get to an accident scene… we work with the fire service whom we call after accidents where the victims are entangled and trapped by motor vehicle wreckage, also when there is leaking fuel around the scene of an accident…bystanders. (Ambulance service)

This network of stakeholder interaction shows the complexity needed for transforming the constantly revised cycling policies into sustained safe cycling.

Importance of Accurate Data

Regarding data source, the VIA database was of common interest to the Municipality, Cycling Union, and SWOV:

…our most important source of data is from VIASTAT. (Municipality)

… The 70% drop was due to a reduction in registration, as can be seen from the national summary of VIA. (Cyclists’ Union)

… SWOV is also involved in the STAR (Smart Traffic Accident Reporting) initiative of VIA… (SWOV)

The VIA database depends on the police and insurance companies for its data. Some stakeholders highlighted that a drop in the number of accidents, after 2009, was due to under-reporting, a situation that affects the accuracy of the database.

The reduction between 2009 and 2010 is due to a reduction in the registration of accidents… data was better 10 years ago than it is now because the police collected data on all accidents… (Municipality)

…Police registration…is weak in the field of injury data collection for all road users, as well as cycling fatalities. (SWOV)

…we stopped registering accidents unless they were severe. Less severe accident victims were referred to their insurance companies instead. (Police)

Other sources of cycling data for the different organizations, in Maastricht, were as follows:

…the media and cyclists who write letters to the organization…we also do surveys, watch and observe traffic situations in the city and infrastructure. (Municipality)

In terms of injuries, the hospital data are better and these are a major source for SWOV - although it becomes less accurate as one gets to the regional levels… (SWOV)

…in the last 2 -3 years we restarted partial registration; recording names, types of injuries, and not many other variables… (Police)

We collect accident data at the scene of the accident, the biodata of the patient, and the kinds of injuries sustained… (Ambulance service)

Every respondent saw an accurate database of cyclists’ numbers, and accident records, as vital for effective planning and monitoring purposes. And the period after 2009, when data collected by the police and insurance companies was reduced, was a source of concern to all the stakeholders.

Investing in Safety

The call to improve and continue investing in cycling infrastructure resonated at the Municipality, Cycling Union, and SWOV.

…the government has to invest more because every dead man on the street is one too many…The design and capacity of infrastructure should be upgraded as the number of cyclists increase…Educational programs should be emphasized…Data collection can be improved if people involved in accidents can input the information directly into the VIA database… (Municipality)

…we should do more for the safety of the elderly cyclist…make the infrastructure more friendly for them…there should be cycling classes for the young… (Cyclists’ Union)

Removing obstructions, and making people wear helmets should reduce cyclist-only accidents… Maastricht should have better data collection initiatives using innovative computer applications and automatic data collection… add data on the number of cyclists and bicycle types in Maastricht. (SWOV)

…make the insurance companies contribute more to the collection of cycling accident data. (Police)

There is the need for the use of protective gear like helmets and reflectors, and the promotion of their use in the young and old... (Ambulance service)

Informant’s Opinions on Quantitative Results

The opinions of the different stakeholders on important phase one findings can be seen in Table 3.

**Table 3:** Opinions of major stakeholders on important trends observed in the quantitative phase of the study SWOV: Institute for Road Safety Research; UM: University of Maastricht

	Municipality	Cyclist Union	SWOV	Police	Ambulance unit
Sex (and injury disparity)	…females are more careful than males and less accident-prone…males are stronger and have better reflexes…	…males have higher risk behaviors…and are also involved in amateur cycling races outside Maastricht, but in this city, it's likely to be due to drunk riding… there are more elderly women than men and this explains why females are seen to have a higher propensity for injuries…	…if more males cycle in Maastricht, that would make the pattern logical. Also, there is a lot of bike racing by males in that part of the Netherlands… SWOV found out that, at the national level, females are injured more often because of their higher proportion in the older age groups…	I think women are much more careful riders.	… Males are more careless. Also, they tend to indulge in cycling races… females are more prone to injury probably because they aren’t physically as strong as men…
Age	… age-groups below 12 should have a higher rate of injuries…it’s true that elderly people are more prone to injuries…	10-20-year-olds are involved in accidents more often because most cyclists are in that age bracket …in the Netherlands, children go to school by bicycle, during rush hours…cyclists in that age group are also not adept at cycling yet…	30-40 years have fewer cycling accidents because they commute more with cars than bicycles…the younger age groups are also more accident-prone because they party a lot on weekends…	…at the age bracket 10-20, a lot of people go to school and they do so during the rush hours. The rise for those over 50 years comes from the use of E-bikes…	…we see a lot of accidents among young students going through and fro school and university students who use old bicycles and sometimes ride while drunk… We mostly treat elderly people with serious injuries, while the younger age groups are more likely to have minor gashes…
Years	The 70% reduction between 2009 and 2010 is due to a reduction in the registration of accidents…the increase from 2013 was most likely due to E-bikes, not from an improvement in the quality of data collection…	…the decrease after 2009 is due to a reduction in registration rates.	…a 70% drop is likely due to registration bias…reporting bias may be the problem. At the national level, injury counts are going up strongly not reducing…	…probably because in 2010, the police stopped registering non-severe accidents …but nationally there is an increase in cycling accidents due to the use of E-bikes…	
Days	…Thursday more than Friday: probably due to being tired at the end of the working week, but the low weekend rates are explained by the low traffic volumes during that period…	Less traffic and cycling on weekends explain the low numbers…	A SWOV research showed that there were extremely high BAC levels in – Groningen and The Hague - two university cities on Thursday and Friday nights …	…weekends have fewer cyclists and other vehicle users. The start of the week comes with alert road users but by the end of it, road users are tired and prone to make mistakes…	…Thursday and Friday are associated with people coming into Maastricht for a weekend of sports cycling. Also, traffic volume is larger during the weekdays but smaller on weekends…
Time period	… 9 am-12 pm and 4pm-6 pm has lots of traffic jams in Maastricht with slow drivers, but between 12- 4 pm there are often none so that with more space cars can drive faster, increasing the chance of bicycle and car accidents…	I would expect more accidents during rushing hours, but the time ranges used for analysis are not equal…		… likely as there is a lot of travel volume during the periods 9 am-12 pm and 4-6 pm.	The period from 11 am is when our day starts to get busy. We remain busy with traffic emergencies until about 6 pm…
Vehicles involved	…cyclists and car accidents are commonest, and produce the highest number of severe cycling accidents…	There are many more cycling-only/ cycling + paedestrian / cycling + cycling accidents than reported because they are usually not very serious. Accidents with buses/lorries are rare because these involve professional drivers who rarely hit cyclists…	Nationally cyclist-only accidents are the most registered because there is a bias towards such accidents…resulting in seemingly fewer cyclists-only accidents…	…heavy vehicles don’t move fast and cycling accidents involving them often happen on shared roads, due to the phenomenon of blind spots. But smaller vehicles move faster, and are more in number so that there are more accidents with them …	…we see more cyclist only accidents, and then bicycles with other 2 wheelers than we see bicycles and motor cars …
Districts		Northeast is dangerous because it is in an industrial belt and is near the motorway with incoming traffic, and Duurzaam veilig isn’t well implemented in the area….not much traffic in Maastricht central/Southwest districts, hence the lower numbers…	…if the Northeast is an area with a lot of intersections and high speeds then it would be expected to have more cycling accidents…	…depends on the geography of Maastricht. To the east (north and south) there a lot of children from the countryside who come to Maastricht to school…	
Nature of road	in Duurzaam veilig if there is a mismatch then an accident should occur...this would explain why more cycling accidents occur on shared lanes than on cycling lanes…intersections will have higher accidents, and roundabouts are expected to be safer…	Roundabouts are safer than intersections, though this improvement is seen more with cars…more accidents occur on shared roads because visibility is poorer for drivers on them…		Most roundabouts retain separation of cycling lanes from other traffic users while intersections do not… separation of traffic is good…	
Seasons and weather	Fewer people cycle in wet weather…	It rains only 6% of the time, so to have 12% of cycling accidents in wet weather shows it is relatively high. Visibility is reduced when it rains… Also, cobblestones in the city become slippery.		in autumn leaves fall on the ground, and together with rainwater make the road very slippery and accident-prone for cyclists.	Autumn is dangerous because the leaves on the ground mix with the rains and become very slippery.
Severity of injuries	…accidents involving heavy vehicles are the most severe…	…poor reporting means there should be a higher proportion of accidents with no injuries…also the reduction in injuries is due to poor reporting….	…international definition of severity uses MAIS (maximum accident injury scale – MAIS)… MAIS 3+ is the international definition of severe injury…in the Netherlands, it is MAIS 2, plus one night in hospital…	…non-injurious accidents should be higher…	
Causes of injury	E-bikes are a new safety concern because they are faster than normal bikes (10-15km/h), and have to use the same lane; which is just 1.5-2.5 meters wider…	…refusal to grant priority is mostly what happens at intersections…the increased variety of bicycles results in speeding differences on lanes…			
Others		Maastricht is safe… young people “fall and get up” so the probability of them being injured should be less… the number of cyclists is increasing…this increase is most likely due to students in UM			I think Maastricht is really a safe place to cycle, unlike Amsterdam with its large numbers of cyclists and people who flaunt traffic rules…

## Discussion

This study set out to assess the trend of cycling accidents in the city of Maastricht from 2001 to 2015 and to understand the opinions of major stakeholders on the quality of cycling data, the quantitative findings from the study, and future policymaking. No such study had yet been done in the city of Maastricht.

It concluded that major improvements in the database used by the city for planning and monitoring its cycling safety operations are a priority. A reduction in the number of accidents across the fifteen-year period might have been interpreted to mean Maastricht city was succeeding with cycling safety; however, major stakeholders sounded a note of caution by ascribing this reduction to a decrease in the quality of data collection from 2009, instead. All the stakeholders, thus, concurred on the need to strengthen and modernize data collection and proffered various ways to achieve this.

The same conclusions were drawn for an observed decrease in the number and risk of getting an injury across the years was observed, which was at variance with what was seen at the national level where a steady increase in the number of injuries from cycling accidents was a source of concern [34]. This shows that the importance of reliable and accurate data cannot be overemphasized as it helps to “identify road safety problems, risk factors, and priority areas” [35].

Most accidents we analyzed were between cyclists and motorcars or vans with increased severity amongst cyclist-only and bicycle-pedestrian accidents, as well as with bicycle and two-wheeler accidents. However, a number of studies show that cyclist-only accidents are the most prevalent form of cycling accidents in the Netherlands. These studies also point out that accidents involving motorized vehicles often result in more severe injuries and fatalities than do single bicycle counterparts [13,34,36-38]. Understanding the types of accidents experienced by cyclists in the city and the nature of their severity will be important in future infrastructural design; indeed, our respondents highlighted various links between the nature of cycling infrastructure in an area and the types of accidents happening there.

Another observed trend was that most of the cycling accidents involved people who were young and males. On the other hand, more accidents with adverse outcomes (injuries or death) were seen amongst elderly and female cyclists. Statistics by SWOV, at the national level, agreed with this trend [34]. Our respondents ascribed the situation to the inexperience of young cyclists, the larger number of them who cycle, and the high use of old bicycles by university students. They also opined that the carefree nature of male cyclists and their proneness for riding while drunk played a role; as did the delicate physical nature of females and the elderly, most of whom ride with no form of protection (common in the Netherlands). Risky behaviors like using electronic devices while cycling is also more likely amongst the young, with old Dutch studies showing phone use as a culprit in 3-4% of cycling accidents with injuries [22,39].

Intersections are the most dangerous points on the road for a cyclist; roundabouts being the safest. In a lot of cities in the Netherlands, there is a push towards converting intersection points to roundabouts [13,34,35], a view shared by some of our respondents who explained that in roundabouts, separation of traffic was usually maintained with bicycles kept away from motorized traffic, a situation that is harder to replicate at an intersection. However, a note of caution on the application of these data was sounded by a respondent, who said some of the current research on the subject showed the positive effects were majorly for cars.

Furthermore, designing infrastructure properly does not guarantee safety alone. Maintaining them and educating users on their safe use are also important factors. Road surfaces that are uneven (with potholes or cobblestones, for example), unclean, and improperly marked tend to be more accident-prone [13]. In a similar vein, the study showed that autumn and wet weather made cyclists more prone to accidents and injuries. Respondents linked the slippery nature of roads to the wet fallen leaves of autumn and wet cobblestones during rains or winter.

Maastricht central and the Southwest, combined, had fewer accidents and adverse accident outcomes. The unique road design espoused by Sustainable Safety Vision (Duurzaam veilig) is credited for this situation; as the area has benefited from “street hierarchy” and separation of traffic which reduce the exposure of cyclists to motorized vehicles. These are also areas with a high number of residential houses and a road network designed for such. Therefore, this shows that the policy of Sustainable Safety and the bicycle plan of the city, which follows its principles, can succeed in improving safety if well implemented [8,40].

Limitations

The study had a three-month limit which resulted in fewer stakeholders being interviewed, also, because the data had to be manually transferred from VIA’s database to SPSS, imputation errors may have occurred. Furthermore, a change in the police protocol for recording cycling accident data in Maastricht, from 2010, resulted in a greater emphasis on serious accidents a possible source of instrumentation bias and a threat to the internal validity of the study. Finally, by applying multidimensional qualitative coding to one-dimensional quantitative codes [41,42], the richness of the qualitative data and the precision of the quantitative data will both have been affected at the point of complementary integration.

## Conclusions

The study tried to understand the nature of cycling accidents in Maastricht using the VIA database and major stakeholder analysis. And we conclude that major improvements in the database used by the city for planning and monitoring its cycling safety operations need to be made. Also, most stakeholders agree that for Maastricht to become safer for cyclists than it already is, there needs to be greater coordination and harmony between the various stakeholders.

Therefore, the city of Maastricht should strive to improve its cycling accident database by utilizing other data sources like those of hospitals, coroners, or ambulance services, in addition to VIA. The new STAR partnership between VIA, the Police, and the insurance companies, promises to improve the accuracy of this database, but VIA should also find a way to incorporate direct reporting by the individual cyclist. This will go a long way to reduce underreporting by those cyclists who do not sustain grievous injury to self or damage to property, who may therefore feel unobligated to make contact with the police or insurance company.

VIA should also improve the consistency of entries, and eliminate variables that are difficult to collect. Also, the city should seek to make its own cycling accident database easy to access in the future, by providing them in formats that can easily be read using different statistical software programs (like SPSS). Furthermore, when the quality of data collection has been improved, it will be helpful to include population distribution figures for each district, cycling distance traveled, and cycling volume figures to enable the accurate calculation of prevalence and incidence rates.

A more detailed stakeholder analysis prior to finalizing the new cycling safety policy of the city is recommended in order to arrive at an understanding by all cycling stakeholders in a way that would make the new policy easily implementable. The stakeholder analysis should also look into the possibility of educating young cyclists on the benefits of helmet use and the avoidance of riding under the influence of alcohol or drugs. But this will not be easy as the policy process is complex and interactive - both at the formulation and implementation levels - with many groups to engage with. The World Health Organization tried to classify the various road safety groups of importance into providers, enforcers, and users. In the case of Maastricht, applying this classification might look like this: Providers will be the provincial government and the municipality’s traffic safety, management, and transport arms; Enforcers will be the police, educators, media, SWOV, ROVL, health professionals and the ambulance service; and users will be the individual cyclists and their advocacy group the Cyclists’ Union.

The stakeholder analysis may also want to consider which policy process will best suit the city’s needs. A top-down pattern, in which there is a clear division between those who formulate policy and the implementers, might seem more acceptable to the Providers, while a bottom-up approach will favor Enforcer and User groups as it gives them “some discretion to reshape the dictates of higher levels in the system”. In contemporary Public Health, the bottom-up approach is most preferred as it takes into account local influences without necessarily negating the role of the policymaker.

The North-Eastern and South-Eastern parts of the city will require that more attention be paid to their cycling infrastructure. We share the views of some of our respondents that the districts can be made safer if they become more compliant with the Sustainable Safety Vision plan (Duurzaam veilig). Upgrading existing infrastructure across the city must continue to occur with the changing times. For example, converting more intersections to roundabouts, replacing cobblestone streets with less slippery surfaces, and making cycling lanes wider and more “forgiving” so that they become more suitable for the elderly and E-bikes. Finally, there are a number of studies on ways to make motorcars and heavy vehicles less dangerous to cyclists, in the event of a collision. Introducing some of the recommendations of such studies into the city’s bicycle plan will be helpful in improving the safety lot of the city’s cyclists.

## Appendices

QUESTIONNAIRE

1) About your organization.

a. What is your organization’s contribution to cycling safety in Maastricht and the Netherlands?

b. What are the current policies, programs, and action plans the organization is guided by?

c. How do you feel about these policies, programs, and action plans (national government, or other bodies?)

d. How are the organization’s operations, relating to cycling safety, carried out?

2) About other stakeholders you work with.

a. What is the relationship between your organization and other stakeholders in cycling safety?

b. What advice can your organization give other stakeholders?

3) Data collection on cycling accidents in Maastricht.

a. How does your organization help collect data on RTAs involving cyclists?

b. Do you think there are problems with data collection? If so, what is being done about these problems?

c. How does the organization use data it collects for its planning and operations?

At this point, please go through the summary of results from the Quantitative data analysis, and then continue.

d. In your view, is there an explanation for the different trends seen?

e. Are there specific questions on the data you want us to address?

f. Are these findings in any way useful to your organization, and if so, how?

4) Do you have any individual ideas and contributions to the study and cycling safety in general?

a. The amount of serious accidents in the Netherlands is already quote low - do you think there is still room for improvement, and would the effort be a worthwhile investment?

Thank you so much for your time!!!!
